# Expression of HIV receptors, alternate receptors and co-receptors on tonsillar epithelium: implications for HIV binding and primary oral infection

**DOI:** 10.1186/1743-422X-3-25

**Published:** 2006-04-06

**Authors:** Renu B Kumar, Diane M Maher, Mark C Herzberg, Peter J Southern

**Affiliations:** 1 Department of Microbiology, University of Minnesota, Minneapolis, MN 55455, USA; 2 Department of Diagnostic and Biological Sciences and the Mucosal and Vaccine Research Center, University of Minnesota, Minneapolis, MN 55455, USA

## Abstract

**Background:**

Primary HIV infection can develop from exposure to HIV in the oral cavity. In previous studies, we have documented rapid and extensive binding of HIV virions in seminal plasma to intact mucosal surfaces of the palatine tonsil and also found that virions readily penetrated beneath the tissue surfaces. As one approach to understand the molecular interactions that support HIV virion binding to human mucosal surfaces, we have examined the distribution of the primary HIV receptor CD4, the alternate HIV receptors heparan sulfate proteoglycan (HS) and galactosyl ceramide (GalCer) and the co-receptors CXCR4 and CCR5 in palatine tonsil.

**Results:**

Only HS was widely expressed on the surface of stratified squamous epithelium. In contrast, HS, GalCer, CXCR4 and CCR5 were all expressed on the reticulated epithelium lining the tonsillar crypts. We have observed extensive variability, both across tissue sections from any tonsil and between tonsils, in the distribution of epithelial cells expressing either CXCR4 or CCR5 in the basal and suprabasal layers of stratified epithelium. The general expression patterns of CXCR4, CCR5 and HS were similar in palatine tonsil from children and adults (age range 3–20). We have also noted the presence of small clusters of lymphocytes, including CD4^+ ^T cells within stratified epithelium and located precisely at the mucosal surfaces. CD4^+ ^T cells in these locations would be immediately accessible to HIV virions.

**Conclusion:**

In total, the likelihood of oral HIV transmission will be determined by macro and micro tissue architecture, cell surface expression patterns of key molecules that may bind HIV and the specific properties of the infectious inoculum.

## Background

Oral exposure to HIV infectivity is known to occur in mother-to-infant transmission by nursing [1-3] and for participants in receptive oral intercourse [4-7]. HIV virions and/or HIV infected cells are shed in body fluid released by the donor and mucosal surfaces in the oral cavity of the recipient are transiently coated with HIV infectivity. Many different mechanisms exist to protect mucosal surfaces from HIV infection in the oral cavity [8,9] but if the epithelial barrier is damaged or if virions invade the epithelial cell layers then infectious HIV virions may readily come into contact with susceptible CD4^+ ^T cells [10]. Several studies using the simian immunodeficiency virus (SIV)/rhesus macaque model have established that atraumatic oral SIV inoculation can result in primary SIV infection in palatine tonsil, followed rapidly by systemic SIV infection [11-14]. Direct analysis of tissue from HIV-infected patients has also implicated palatine tonsil as a reservoir and replication site for HIV [15-17]. In an attempt to gain further insight into the process of oral transmission, we and others have created *ex vivo *organ culture systems with human palatine tonsil that recapitulate HIV exposure to varying extents [10,18-21]. These studies have provided valuable new information concerning the cellular and molecular events that support oral HIV transmission but many fundamental questions remain unresolved.

The external surface of the human palatine tonsil is primarily covered by a stratified squamous epithelium where the most external terminally differentiated cells are continually sloughed away and replaced by proliferation of cells displaced upwards from the basal cell layer. In contrast to the proximal oral mucosa, the palatine tonsil surface is notable for the presence of openings that provide access into the tonsillar crypts. The surfaces of the crypts are lined by reticulated epithelium and the actual barrier between the lumen of the crypt and intraepithelial lymphocytes may only be one epithelial cell layer thick. The unique cellular composition of reticulated epithelium, where epithelial cells, leukocytes and stromal cells are all situated in close proximity, has been associated with the ongoing process of antigen sampling in the oral cavity [22,23]. In the rabbit [24], there is direct evidence for the presence in tonsillar crypts of M-like cells that correspond to the M (Microfold) cells found overlying accumulations of lymphoid cells (Peyer's patches) in the intestine but the presence of M cells in the human palatine tonsil has not been confirmed definitively [25,26]. In the context of oral HIV transmission, the cellular composition and microarchitecture of the mucosal epithelial surfaces can be projected to have a major impact on whether exposure to HIV will actually progress to the establishment of primary HIV infection in the recipient.

In HIV/AIDS patients, the vast majority of the HIV infection is confined to the CD4^+ ^subset of T cells in lymphoid tissues [15,27,28] and the CD4 molecule was identified more than twenty years ago as the primary receptor for HIV infection [29]. In subsequent studies, a connection was established between HIV infection and virus recognition of co-receptors expressed on the target cell surface. The principal co-receptors, CCR5 and CXCR4, like the CD4 primary HIV receptor, are normal T cell surface proteins with key roles in immune signaling and T cell function, as reviewed in Berger *et al*. [30]. Epithelial cells that are susceptible to HIV infection have been reported to express CXCR4 and CCR5 [31,32] but other studies have not succeeded in establishing HIV infection in cervical and prostate epithelial cells [33]. In a number of cases, however, HIV infection has been detected in cells with low to undetectable levels of CD4 expression and these observations prompted a search for alternate primary HIV receptors. To date, heparan sulfate proteoglycan (HS) and galactosyl ceramide (GalCer) have been identified as cell surface macromolecules that can support HIV infection in the absence of CD4 recognition by gp120 projecting from the envelope of HIV virions [34-36]. Additional interactions between virions and mucosal surfaces may be supported by host cell surface components that are routinely incorporated into HIV envelopes [37,38]. For example, the presence of ICAM-1 on the surface of HIV virions allows recognition by the physiological receptor, LFA-1, expressed on the target cell surface [39,40]. At the other extreme, binding of retrovirus particles to target cells has been demonstrated to occur in the complete absence of virus envelope constituents [41,42]. It is therefore apparent that a spectrum of interactions can occur between HIV virions and exposed mucosal surfaces and that both the properties of the inoculum (cell free HIV virions and/or cell associated infectivity in the form of HIV-infected cells) and the characteristics of the exposed surface will contribute to the overall susceptibility to HIV infection. In this study, we set out to document epithelial cell expression patterns for key cell surface molecules, implicated directly and indirectly in HIV virion binding to mucosal surfaces to account for the extensive binding of HIV virions to human palatine tonsil that we have previously reported.

## Results

### Immunocytochemical definition of epithelial cell surfaces in human palatine tonsil

**Figure 1:**
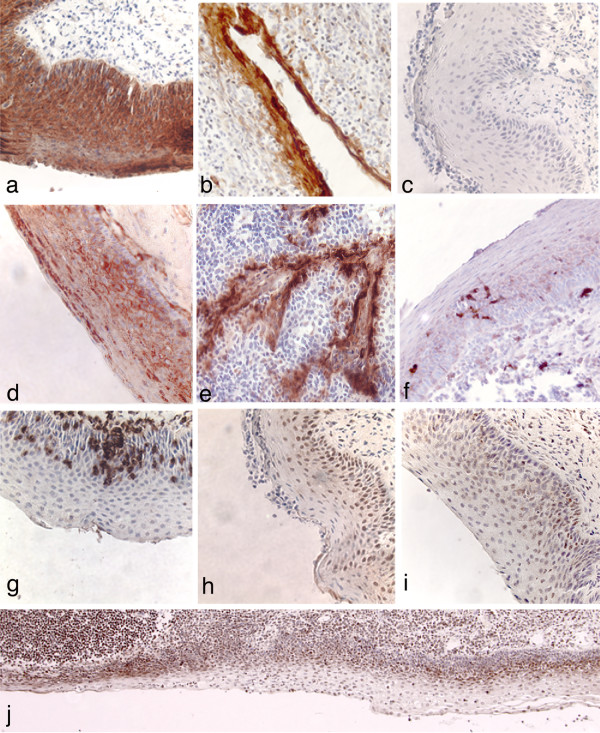
Immunocytochemical detection of cell surface macromolecules expressed on stratified squamous epithelium and reticulated cryptal epithelium in human palatine tonsil. Tissue sections were incubated with primary antibodies as indicated below. Positive cells were identified with biotinylated secondary antibodies and streptavidin-peroxidase conjugates and are stained brown. All sections were counterstained with hematoxylin. a: cytokeratin-stratified squamous epithelium; b: cytokeratin-cryptal epithelium; c: control mouse antibody; d: HS; e: GalCer-cryptal epithelium; f: S100-dendritic cell marker; g: CD3; h: CXCR4; i: CCR5; j: CXCR4 – showing variability in the distribution of CXCR4 positive cells and the reduced thickness of stratified epithelium overlying a follicle at the lower left side. Note that in this large stretch of epithelium, an occasional CXCR4^+ ^cell may be a dendritic cell but based on Fig 1f, the overall abundance of dendritic cell is very low. Original magnification a-i: ×400; j: ×100.

Antibodies directed against representative epithelial cell antigens were used as internal controls to establish the specificity of the immunocytochemical staining procedures for the panel of tonsils studied. Epithelial cells in stratified squamous epithelium and cryptal epithelium were identified with a polyclonal rabbit anti-cytokeratin antibody (Figure 1a, b). Epithelial cells were independently detected with a mouse monoclonal antibody directed against Hsp27 ([43], Table 1). The anti-Hsp27 antibody also bound to endothelial cells in all tonsil sections and a subset of T cells ([44], Table 1). A polyclonal antibody directed against interleukin-8 (IL-8) was used to evaluate the activation status of epithelial cells [45,46] and a broad distribution of positive epithelial cells was observed in both stratified squamous epithelium and reticulated epithelium (Table 1). In addition, staining of a subset of T cells, randomly distributed throughout the T cell zone was consistently observed with the anti-IL-8 antibody (Table 1).

**Table 1:** Qualitative immunocytochemical analysis of key cell surface macromolecules expressed on human palatine tonsil.

Tonsil	Age	CXCR4			CCR5			HS			CD4			IL-8			Hsp27		
		
		SE	CE	T	SE	CE	T	SE	CE	T	SE	CE	T	SE	CE	T	SE	CE	T
1	3 F	i	+	i	i	+	i	+	+	-	-	-	+	i	+	i	+	+	-
2	4 F	i	+	i	i	+	i	+	+	-	-	-	+	+	+	i	+	+	-
3	5 F	i	+	i	i	+	i	+	+	-	-	-	+	i	+	i	+	+	i
4	6 M	i	+	i	i	+	i	+	+	-	-	-	+	i	+	i	+	+	i
5	15	i	+	i	i	+	i	+	+	-	-	-	+	i	+	i	+	+	i
6	17	i	+	i	i	+	i	+	+	-	-	-	+	+	+	i	+	+	-
7	17 F	i	+	i	i	+	i	+	+	-	-	-	+	+	+	i	+	+	i
8a	18 F	i	+	i	i	+	i	+	+	-	-	-	+	+	+	i	+	+	-
8b	18 F	i	+	i	i	+	i	+	+	-	-	-	+	+	+	i	+	+	-
9	20 F	i	+	i	i	+	i	+	+	-	-	-	+	+	+	i	+	+	i

Frequency		9/9i	9/9+	9/9i	9/9i	9/9+	9/9i	9/9+	9/9+	9/9-	9/9-	9/9-	9/9+	4/9i 5/9+	9/9+	9/9i	9/9+	9/9+	5/9i 0/9+

SE: stratified squamous epithelium; CE: cryptal epithelium; T: T cells; +: uniform strong positive expression; i: intermittent strong positive expression; -: no detectable expression. SE and CE were strongly and uniformly positive with a polyclonal antibody to cytokeratins; there was no detectable expression of cytokeratins in T cell populations (data not shown). Complete T cell populations were identified with anti-CD3 and most of the T cells were CD45RO positive (data not shown). Results for 8a and 8b were obtained from two independent tissue blocks derived from the same piece of tonsil. Specific staining conditions and optimized antibody dilutions are presented in Methods. In the "Age" column, M and F refer to male and female respectively, where known.

We also examined the distribution of ICAM-1 and LFA-1 on tonsil epithelial surfaces because this ligand/receptor interaction has been linked to HIV virion binding to cell surfaces [37,47]. ICAM-1 expression was localized exclusively to the reticulated cryptal epithelium where the positive cell populations included epithelial cells, lymphocytes and endothelial cells. No ICAM-1 expression was detected on or within stratified squamous epithelium. Only weak staining was observed in reticulated epithelium with an antibody directed against LFA-1 (data not shown).

### Expression of the alternate HIV receptors heparan sulfate proteoglycan and galactosyl ceramide

HIV attachment to cell surfaces is known to involve recognition of proteoglycans that are widely distributed on the surfaces of different cell types [34,35,42]. We detected expression of HS on all cell layers comprising tonsillar stratified epithelium, although in many areas we noted elevated HS expression in the suprabasal layers of squamous epithelial cells (Figure 1c, d, Table 1). Within tonsillar crypts, the entire epithelial surface was lined with cells expressing HS. Epithelial cells expressing GalCer were detected within the tonsillar crypts (Figure 1e) but there was extensive variability between tonsil donors.

### Cellular invasion of stratified squamous epithelium

In addition to the prototypical content of epithelial cells in varying stages of differentiation, we routinely observed migrating dendritic cells (DC), macrophages and small foci of invading T cells within stratified squamous epithelium. The numbers and distribution of DC, macrophages and T cells were highly variable across a stretch of stratified epithelium (Figure 1f, g, data not shown for macrophages) but each of these cell types could be detected within stratified epithelium for all of the tonsils examined.

### Expression of HIV co-receptors CXCR4 and CCR5

Several previous studies have reported expression of the principal HIV co-receptors, CXCR4 and CCR5, on cultured epithelial cells [32,48,49] and that oral epithelial cells are susceptible to HIV infection *in vitro *[31,50]. A detailed evaluation by fluorescence activated cell sorting (FACS) of co-receptor expression on tonsil cell suspensions provided valuable information relating to lymphocyte populations [51] but, by gating on populations of single cells of defined size, this study would probably have excluded epithelial cells. We therefore set out to establish expression profiles for CXCR4 and CCR5 on tonsillar epithelial surfaces using palatine tonsil sections. Epithelial cells expressing either CXCR4 or CCR5 were detected in the basal and suprabasal layers of stratified squamous epithelium (Figure 1h, i) but there was wide variability in the numbers of positive cells across a continuous stretch of stratified epithelium (Figure 1j). Given the observed low frequency of dendritic cells (Fig 1f), which also may express CXCR4 and/or CCR5 [52,53] we concluded that the co-receptor positive cells could not be explained in terms of dendritic cells within the stratified squamous epithelium. This point was explored directly in double labeling experiments (see below). Within the tonsillar crypts, the reticulated epithelium showed extensive expression of CXCR4 and CCR5. The general characteristics of CXCR4 and CCR5 expression by epithelial cells were consistent for tonsils obtained from nine different subjects, representing tissue donors 3–20 years old (Table 1).

### Distribution of T cell subsets in human palatine tonsil

**Figure 2:**
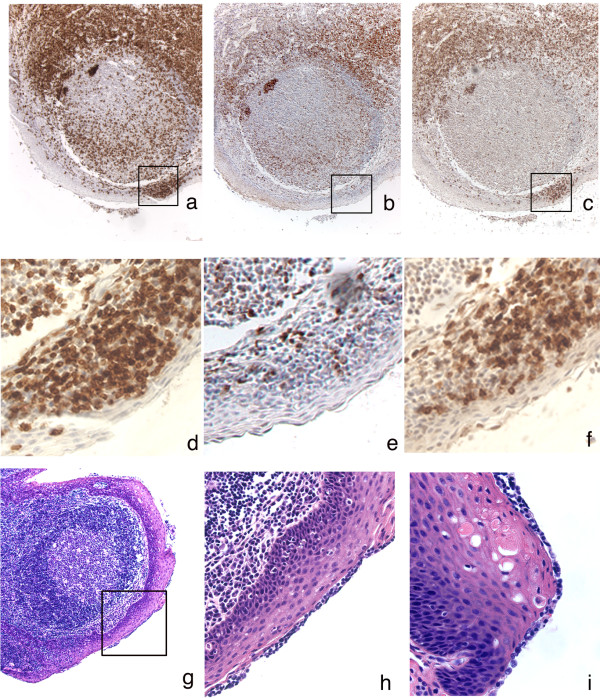
T cell distribution in proximity to the luminal surface of human palatine tonsil. Tissue sections were incubated with primary antibodies as indicated below. Positive cells are stained brown. Sections were counterstained with hematoxylin. a: CD3; b: CD4 – large irregular shaped CD4^+ ^cells within follicles are macrophages; c: CD8; d: CD3; e: CD4; f: CD8 – d, e and f represent enlargements of the regions enclosed within boxes in a, b and c respectively; g: H&E staining of tonsillar epithelium; h: enlargement of region enclosed within the box in g; i; independent tonsil section stained with H&E to show surface lymphocytes. Original magnification a, b, c, g: ×100; d, e, f, h, i: ×400.

**Figure 3:**
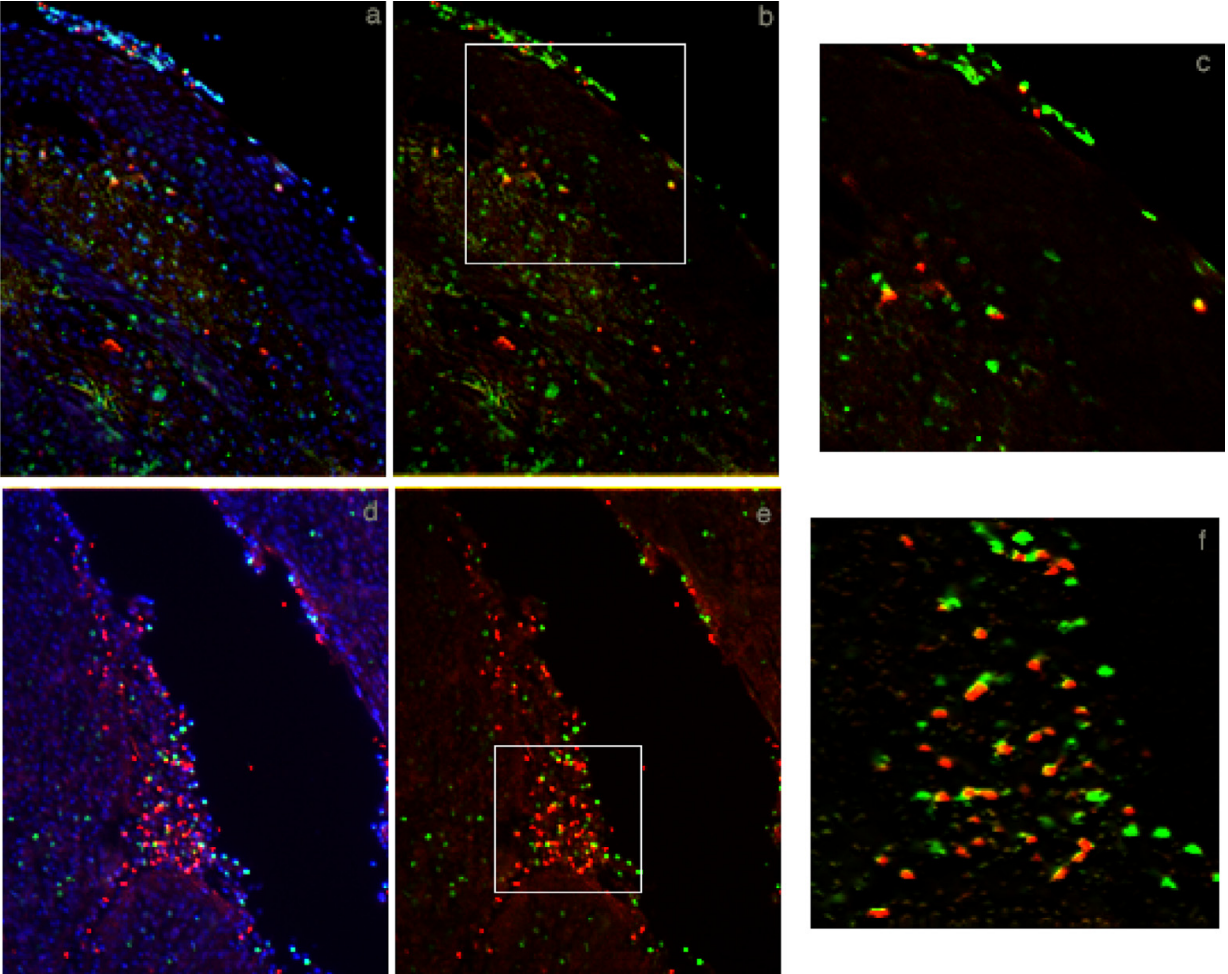
Double label immunofluorescence detection of CXCR4 or CCR5 co-receptor positive T cells at tonsillar epithelial surfaces. Thin sections were incubated with primary antibodies and species-specific fluorescently conjugated secondary antibodies as indicated. All sections were stained with DAPI (blue) to identify cell nuclei. a: CD3 (green) + CCR5 (red): DAPI overlay; b: CD3 (green) + CCR5 (red): no DAPI overlay; c: enlargement of the region enclosed in the box in b; d: CD3 (green) + CXCR4 (red): DAPI overlay; e: CD3 (green) + CXCR4 (red): no DAPI overlay; f: enlargement of the region enclosed in the box in e. Cells that are positive for both markers appear yellow; a-c depict stratified squamous epithelium, d-f depict reticulated cryptal epithelium. Original magnification a, b, d, e: ×100.

In the course of processing tonsil sections with antibodies to CXCR4 and CCR5, we identified T cell subsets that expressed these surface antigens. Co-receptor positive T cells were principally detected in the T cell zones in palatine tonsil (extrafollicular areas). Systematic analysis of T cell distribution was performed with a panel of T cell specific antibodies: CD3 (marker for all T cell subsets), CD4 (helper T cells and macrophages; note the absence of any detectable CD4 expression on epithelial cells), CD8 (cytolytic T cells; Figure 2a–f, Table 1). Both CD4 and CD8 positive T cells were located primarily in extrafollicular areas, and the majority of these T cells expressed the CD45RO activation marker (data not shown). Some T cells, predominantly CD4^+ ^cells, were consistently detected within B cell rich follicular structures, as would be expected in lymphoid tissue involved with ongoing immune responses (Figure 2b, c). Another indicator of immune activation in palatine tonsil was revealed by the identification of clusters of T cells that had invaded the basal and suprabasal layers of stratified epithelium. These invading cells were a mix of approximately 25% CD4^+ ^and 75% CD8^+ ^T cells, with some of these T cells expressing either CXCR4 or CCR5. A series of double label immunofluorescence experiments were performed to confirm that tonsillar stratified squamous epithelium contained both CD3^+ ^CXCR4^+ ^or CCR5^+ ^T cells (Figure 3) and cytokeratin^+ ^CXCR4^+ ^or CCR5^+ ^epithelial cells (data not shown).

In addition to finding CD4^+ ^and CD8^+ ^T cells within epithelial layers, we also detected T cells at the luminal surface of otherwise undisturbed stratified squamous tonsillar epithelium. Retrospective analysis of representative hematoxylin and eosin (H&E) stained slides from randomly selected tonsils indicated that equivalent surface accumulations of T cells could be found in 21 of 30 tonsil samples examined (Figure 2g–i). We were very concerned about artifactual trapping of lymphocytes at the tissue surface either because the tissue pieces had been fixed while still covered with a film of blood or because of relocation of tissue fragments during sectioning. However, the surface lymphocytes appeared to be enclosed within a membrane and in continuous contact with the underlying epithelial cells, suggesting that the lymphocytes had been naturally present on the tonsil surface prior to the surgery. The visual absence of erythrocytes in these surface accumulations of lymphocytes provided further support for a potentially significant biological role for these surface T cells.

### Epithelial damage and HIV infection of tonsil cells

**Figure 4:**
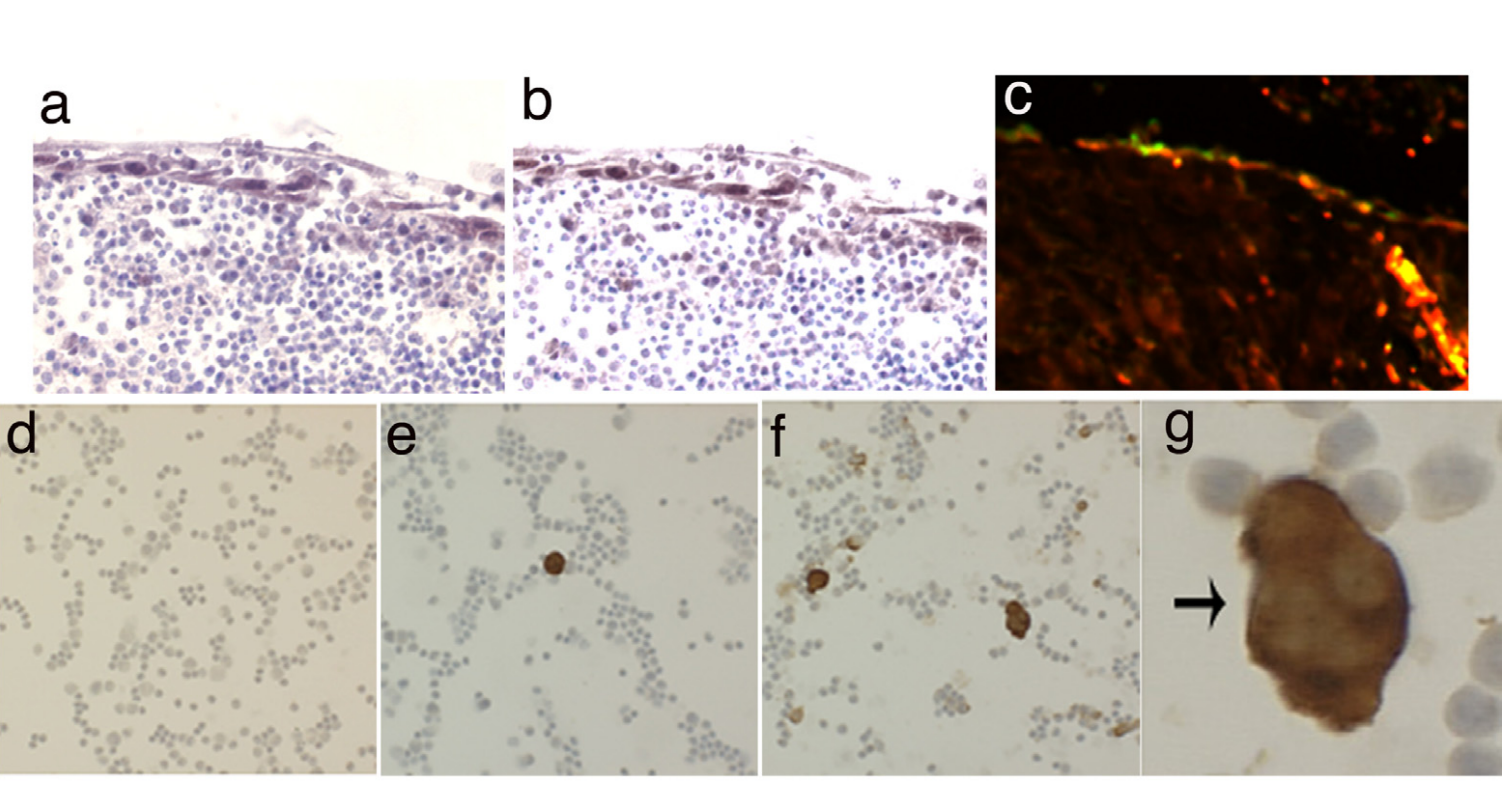
Epithelial damage and repair in *ex vivo *tonsil organ culture and HIV infection of tonsil cells. Small randomly cut pieces of tonsil tissue reacquired an epithelial cell coating during organ culture. Thin sections were incubated with primary antibodies and species-specific conjugated secondary antibodies as indicated. a: CCR5; b: CXCR4; c: CCR5 (red) plus CXCR4 (green), cells that are positive for both fluorescent markers appear yellow. Original magnification a, b, c: ×200. Tonsil cell suspensions were infected with HIV 96–480 patient isolate virus stock, then cells were spotted onto glass slides for immunocytochemical detection of HIV p24 gag: d: day 0, prior to infection; e: day 5; and f: day 10 after infection; g: enlargement from f. HIV infected cells are stained brown; cell nuclei were identified with a hematoxylin counterstain. Original magnification d, e, f: ×100; g: ×400.

In an extreme representation of tonsil damage that would involve complete removal of the protective epithelium, small cut pieces of tonsil tissue were maintained in organ culture and infected with HIV [21]. As noted previously, cut pieces of tonsil in organ culture supported the spontaneous proliferation of epithelial cells and consistently produced an epithelial cell coating, two-four cell layers thick that enclosed the tonsil pieces [19,21]. The epithelial character of the surface coating of cells was confirmed by strong positive staining with antibodies directed against cytokeratins (data not shown). These newly proliferated epithelial cells expressed high levels of CXCR4 and CCR5, and analysis of adjacent tissue sections indicated that many of these epithelial cells could be expressing both CXCR4 and CCR5 (Figure 4a, b). Co-expression of CXCR4 and CCR5 was confirmed by double immunofluorescence labeling (Figure 4c). These surface epithelial cells did not support productive HIV infection, as judged by immunocytochemical staining for HIV p24 gag. However, high-level epithelial cell expression of CXCR4 and CCR5 may be important in the context of HIV virion binding to mucosal surfaces that have been repaired after physical damage.

HIV exposure and infection at a damaged tonsil tissue surface was also simulated using cell suspensions obtained by mechanical disruption of tonsil pieces. These cell suspensions comprised approximately equal mixtures of B and T cells, as judged by immunocytochemical staining of fixed cell spots (data not shown). Experimental exposure of tonsil cell suspensions to cell-free HIV96-480 virions (a primary patient isolate of HIV with dual tropic properties [21]) led to the establishment of widespread HIV infection and multinucleated giant cells were readily visible at day 10 (Figure 4d–g). In this experiment, which equates to complete removal of the epithelial surface, tonsillar lymphocytes were directly accessible to HIV virions and a spreading productive infection was readily established.

## Discussion

**Figure 5:**
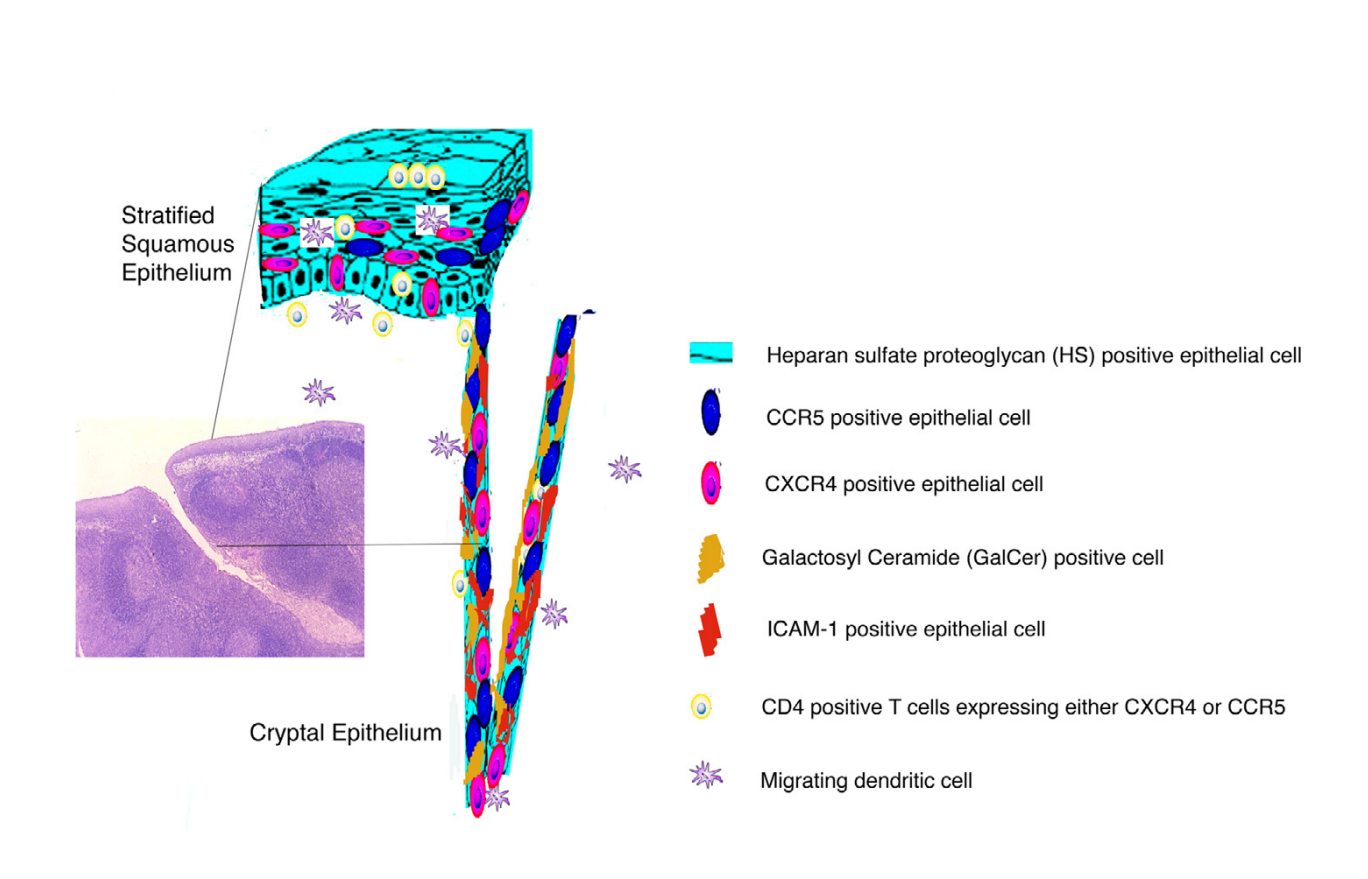
Schematic representation of cell surface macromolecules and migrating cells implicated in HIV binding and uptake. The inset to the left shows a low magnification photomicrograph of a thin section cut through the external surface of human palatine tonsil (H&E; original magnification: ×40). Most of the external surface of the tonsil is protected by stratified squamous epithelium but there is an abrupt transition to reticulated epithelium at the entrance to a crypt. Cell surface molecules that may contribute to HIV virion binding and the cell types expressing these target molecules are depicted in the diagrammatic representations of stratified squamous epithelium and reticulated cryptal epithelium.

We have recently developed a quantitative HIV virion binding assay that documents rapid and extensive binding of HIV virions in seminal plasma to intact mucosal surfaces [10,54]. In the course of these studies, we realized that both micro and macro structural heterogeneity were commonplace at the surface of randomly selected palatine tonsil samples and that surface structural aberrations could have a profound impact on susceptibility to all microbial infections, including HIV. The current study was designed to investigate the molecular basis for HIV virion binding to intact mucosal surfaces by characterizing the expression patterns in palatine tonsil for HIV receptors, HIV co-receptors and other cell surface markers that have been implicated in HIV infection. Based on a comprehensive interpretation of the expression patterns revealed in this study, we conclude that multiple distinct interactions may be supporting HIV virion binding to mucosal surfaces and that the specific molecules involved with binding at any particular site will be directly determined by the precise anatomical location of that site. For example, we have now recognized there are more potential interactions that could support virion binding to reticulated epithelium in the tonsillar crypts than would be immediately available to support virion binding to the luminal surface of stratified squamous epithelium (Figure 5).

When virions in seminal plasma are deposited onto an intact luminal surface of stratified squamous epithelium, the most likely interaction leading to stable binding would appear to involve recognition of HS. If, as has been observed in our previous work [10], virions can penetrate beneath the epithelial surface then there would be the possibility to bind to dendritic cells (Figure 1f, [55-57]) or macrophages present within the epithelium, to bind to CD4^+ ^T cells expressing either CXCR4 or CCR5 (Figures 1g, 2 and 3) that have invaded the epithelium or even to bind to epithelial cells expressing CXCR4 or CCR5 (Figures 1h, i, j, 3 and 4). It is also conceivable that an initial binding event to HS at the luminal surface precedes a cell uptake mechanism (endocytosis or transcytosis) or paracellular transport, allowing the virions to penetrate beneath the luminal surface. These potential interactions involving multiple cell surface macromolecules expressed on several different cell types are presented diagrammatically in Figure 5. There appears to be a large element of chance involved with HIV transmission across mucosal surfaces because epidemiological surveys have revealed that 1 in 200–1000 exposure events are typically associated with male to female heterosexual transmission of HIV [58,59]. This relatively low rate of transmission may be explained, at least in part, by the requirement for random encounters between HIV virions and dendritic cells, macrophages or CD4^+ ^T cells that are transiently located in proximity to the exposed mucosal surfaces. The connection between "chance" and oral HIV infection is also influenced by the morphological characteristics of the exposed surface, including heterogeneity in the thickness of the epithelium, epithelial damage and surface remodeling as a consequence of chronic tonsillar inflammation.

In many of the tissue samples examined for this study we detected accumulations of lymphocytes, including CD4^+ ^T cells at the luminal surface of stratified squamous epithelium (Figure 2g–i). This would imply that virion binding and even primary HIV infection could be initiated at, or very close to the surface of palatine tonsil. It is not necessarily clear how an infection initiated in this manner might spread to other CD4^+ ^T cells but migrating dendritic cells or macrophages could carry the infection back towards the large T cell populations in extrafollicular areas. It is also important to emphasize the variability we have observed in the number of epithelial cell layers that comprise the stratified squamous epithelial barrier (Figure 1j) because contact with large CD4^+ ^T cells pools can be projected to occur much more readily if the distance to be traversed by HIV infectivity is reduced.

If virions in seminal plasma enter into crypts then at least five surface macromolecules – HS, GalCer, CXCR4, CCR5, and ICAM-1 expressed on epithelial cells represent potential binding sites for initial interactions with virions (Figure 5). Furthermore, in reflection of the diverse cell composition of reticulated epithelium, it is likely that CD4^+ ^T cells will be located within 1–2 cell layers of the luminal surface throughout the crypts (Figure 3d–f). The tonsillar crypts have been linked with antigen sampling in the oral cavity and it is conceivable that transcytosis by cryptal epithelial cells with "M-like" properties could place HIV virions in immediate proximity to intraepithelial CD4^+ ^T cells [22,60]. This mechanism of HIV virion uptake would gain substantial credibility with the recognition and functional characterization of M cells in the human palatine tonsil.

The variability observed in the distribution of epithelial cells that expressed CXCR4 and CCR5 within stratified squamous epithelium was unexpected. These findings suggest that surface expression of CXCR4 and CCR5 in epithelial cells may correspond to an early stage in a differentiation pathway but the absence of uniform expression in the suprabasal cell layers remains unexplained. It is interesting to note that similar variability in co-receptor expression patterns was found in populations of primary human epithelial cells, grown out from pieces of palatine tonsil (data not shown). The overall variability revealed in our analyses of epithelial surfaces in palatine tonsil may indicate that the epithelium is in a continuous state of dynamic flux and that the expression patterns of epithelial cell surface molecules are likely to reflect localized influences including repair from physical damage, invasion by inflammatory cells and tissue remodeling as tonsillar lymphocyte populations expand and contract. Because the tissue used in these experiments was removed from patients with tonsillitis, the palatine tonsils analyzed cannot be regarded as strictly normal. However, tonsillar inflammation in the form of a "sore throat" is not uncommon and there is growing recognition of the connection between pre-existing infections and increased susceptibility to exogenous infection [61].

## Conclusion

For the cell surface markers examined in this study, no differences were identified for tissue donors ranging from 3–20 years old. However, we have recognized that structural and functional variability are commonplace at the surface of surgically removed tonsils. Our results reveal a complex expression pattern for HIV receptors, co-receptors, surface adhesion molecules and alternate receptors in stratified squamous epithelium and cryptal reticulated epithelium that individually or collectively could support extensive and stable HIV virion binding. Further insight, leading to the development of a pool of antagonists that effectively blocks virion binding to mucosal surfaces could contribute significantly to a reduction in current rates of HIV transmission.

## Methods

### Tissue collection and processing

Palatine tonsil tissue samples were obtained from routine tonsillectomies performed at the University of Minnesota Medical Center. Tissue donors, or the legal guardian of a child, provided informed consent prior to initiation of the surgery and the protocol to obtain tissue samples had received full IRB approval. All tissues examined in this study were collected from patients with tonsillitis. Tissue pieces were fixed in Streck Tissue Fixative (STF; Streck Laboratories, La Vista, NE) within 1–3 hours of completion of the surgery and then processed by standard methods for paraffin embedding and microtome sectioning. In some instances, tissue pieces were snap frozen in liquid nitrogen for cryostat sectioning. Any tissue with gross macroscopic abnormality was excluded from this study.

### Immunocytochemistry and immunofluorescence detection procedures

Single label immunocytochemistry was performed on paraffin embedded sections (5 μm) and specific antibody binding was detected with biotinylated secondary antibodies and streptavidin-peroxidase conjugates (ABC System; Vector Diagnostics, Burlingame, CA), as described previously [10,21]. Tissues were counterstained with hematoxylin (Sigma-Aldrich, St. Louis, MO) and mounted in Permount (Fisher Scientific, Fair Lawn, NJ). The specificity of staining for individual antibodies was confirmed using either an unrelated isotype control antibody or secondary antibody alone. For some antibodies, where the target epitope was known to be destroyed by paraffin embedding, expression profiles were determined by staining frozen tonsil sections that were fixed in STF immediately prior to use.

Double label detection of target antigens was performed by immunofluorescence staining of paraffin embedded or frozen tissue sections, taking into account the properties of the primary antibodies. In short, after antigen retrieval by citrate buffer, tissue sections were blocked with TNB [0.1 M Tris. HCl pH7.5, 0.15 M NaCl, 0.5% w/v Dupont blocking reagent (Perkin Elmer, Boston, MA)] and 1.5% horse serum followed by overnight incubation with primary antibody at 4°C. Tissue sections were then washed, reblocked and incubated with appropriate secondary antibody (Alexa 568-anti mouse or Alexa 488-anti rabbit conjugates; Molecular Probes Inc, Eugene, OR) for 1 h at room temperature. After dehydration through graded alcohols, the sections were cleared with methyl salicylate (Sigma-Aldrich), mounted in DEPEX (Electron Microscopic Sciences, Ft. Washington, PA) and stored at 4°C until viewed. Fluorescent images were collected with a Zeiss upright microscope equipped with a Spot Camera and motorized stage for high resolution capture of brightfield and fluorescence images and then processed with Adobe Photoshop (Abode Systems Inc, San Jose, CA).

The following antibodies were used at the dilutions indicated: CXCR4 (1:100, clone12G5; BD Pharmingen, San Diego, CA); CXCR4 (1:100, rabbit polyclonal; eBioscience, San Diego, CA), CCR5 (1:750, clone 45549.111; R&D Systems, Minneapolis, MN); Heparan sulfate (1:250, clone F58-10E4; Seikagaku Corp., Tokyo, Japan); CD3 (1:500, rabbit polyclonal; DAKO, Carpenteria, CA); CD4 (1:50, clone IF6; Zymed, San Francisco, CA); CD45RO (prediluted sample, clone UCHL-1; BioGenex, San Ramon, CA); cytokeratin (rabbit polyclonal DAKO); CD54/ICAM-1 (1:50, clone LB-2; BD Pharmingen); CD11a/LFA-1 (1:50, clone G43-25B; BD Pharmingen); DC SIGN (1:50, clone DCN46; BD Pharmingen); S100 (1:5000, rabbit polyclonal antibody; DAKO); IL-8 (1:250, rabbit polyclonal; Santa Cruz Biotechnology, Santa Cruz, CA); Hsp27 (1:250, clone G3.1; BioGenex); GalCer (1:100, clone MAB342; Chemicon, Temecula, CA). The optimal dilution for each antibody was determined empirically in preliminary titration experiments.

### HIV infection of disrupted tonsil

Single cell suspensions from tonsil were prepared by forcing the tissue through a metal tissue sieve. Viable mononuclear cells were further purified by banding on a ficoll gradient and then mixed cell populations, comprised primarily of B and T cells were infected with a low passage, dual-tropic primary patient isolate (HIV96-480; [21]). Infections were routinely performed with HIV stocks diluted to contain 1–5 pg/ml p24 gag. Tonsil disruption, ficoll purification and HIV infection were generally completed within 4–6 hrs of receipt of the tissue into the laboratory. Cell populations were sampled on various days after infection by collecting the culture medium and then cell spots were prepared with washed, concentrated cells. After thorough drying and fixation (15 minutes at room temperature in STF), the cell spots were processed for routine immunocytochemical detection of HIV p24 gag (1:100, clone Kal-1, DAKO).

## Competing interests

The author(s) declare that they have no competing interests.

## Authors' contributions

RBK designed, optimized and performed most of the experiments, interpreted the results and prepared the first draft of the paper.

DMM developed the fundamental procedures to work with tonsil and study HIV virion binding and infection and established fundamental protocols for consistent immunocytochemical staining.

MCH provided critical insight into the design of the experiments, the interpretation of the data and the overall organization of the text.

PJS was responsible for the broad design of the study, processing of the tonsil tissue samples, interpretation and quality control for the immunocytochemistry and completion of the submitted version of the text.

All of the authors made meaningful contributions to the process of revising draft versions of the text.
